# Draft Genome Sequence of a Clinical Isolate of *Streptococcus mutans* Strain HM

**DOI:** 10.1128/genomeA.00826-17

**Published:** 2017-08-17

**Authors:** Yoshio Kondo, Haruka Nishimata, Kiyoshi Hidaka, Tomoyuki Hasuwa, Hiroyuki Moriuchi, Taku Fujiwara, Tomonori Hoshino

**Affiliations:** a Department of Pediatric Dentistry, Nagasaki University Graduate School of Biomedical Sciences, Nagasaki, Japan; b Department of Pediatrics, Nagasaki University Graduate School of Biomedical Sciences, Nagasaki, Japan

## Abstract

We report the draft genome sequence of *Streptococcus mutans* strain HM isolated from a 4-year-old girl with infective endocarditis. The genomics information will provide information on the genetic diversity and virulence potential of *S. mutans* strain HM.

## GENOME ANNOUNCEMENT

Infective endocarditis (IE) is a serious infectious disease commonly caused by non-beta-hemolytic streptococci (1, 2). It has been reported that streptococci isolated from IE patients most frequently belong to the viridans or bovis group (1). *Streptococcus mutans* is a member of viridans streptococci known as a major pathogen of dental caries and a causative agent of IE (3). *S. mutans* strain HM, which was sequenced in this study, was isolated from blood drawn from a 4-year-old girl with a ventricular septal defect who was hospitalized for fever of unknown origin in Nagasaki University Hospital, Japan. The echocardiography showed vegetation near the apex of the tricuspid valve, and she was diagnosed as having IE in accordance with Duke diagnostic criteria (4). Although she had undergone no invasive dental treatment before the onset of IE, detection of the oral streptococci in blood culture implied an intraoral route of infection in this case.

Bacterial cells of *S. mutans* strain HM were inoculated into brain heart infusion broth and incubated under anaerobic conditions. Genomic DNA was extracted using MasterPure Gram-positive DNA purification kit (Epicentre) according to the manufacturer’s instructions. Genome sequencing was performed using an Illumina GAIIx with a 50-bp single-end run. Approximately 7.15 million reads were obtained, and the whole genome was assembled into 186 contigs using Edena (5), with an average coverage of 179×. The draft genome of *S. mutans* strain HM has an approximate size of 2.0 Mb, with a G+C content of 36.7%. The genome sequence was annotated by DFAST (6), and 1,842 coding sequences (CDSs), 31 tRNAs, and 2 rRNA clusters were identified.

The genome of *S. mutans* strain HM has an average nucleotide identity of 99.12% compared to *S. mutans* UA159 (7, 8). Waterhouse et al. reported that 80% of the *S. mutans* CDSs are conserved among 10 strains by microarray hybridization based on the UA159 genome, suggesting that 80% of CDSs are conserved within *S. mutans* strains (9). In this study, HM was found to contain 1,842 CDSs, 1,676 (91.31%) of which are predicted by reciprocal BLAST search analysis to be common to those of UA159, and 7 strain-specific regions including 88 CDSs were unique in comparison with the UA159 genome. Many of the HM-specific genes are foreign genes (i.e., novel genes acquired from the outside of bacterial cells), and it is necessary to study their involvement in the pathogenicity of IE.

### Accession number(s).

The draft genome sequence has been deposited in the DDBJ/EMBL/GenBank database under accession no. BDOS00000000.
